# Design Optimization of Tumor Vasculature-Bound Nanoparticles

**DOI:** 10.1038/s41598-018-35675-y

**Published:** 2018-12-11

**Authors:** Ibrahim M. Chamseddine, Hermann B. Frieboes, Michael Kokkolaras

**Affiliations:** 1 Department of Mechanical Engineering, McGill University, Montreal, Quebec Canada; 2 Department of Bioengineering, University of Louisville, Louisville, KY USA; 3 James Graham Brown Cancer Center, University of Louisville, Louisville, KY USA; 4 GERAD – Group for Research in Decision Analysis, Montreal, Quebec Canada

## Abstract

Nanotherapy may constitute a promising approach to target tumors with anticancer drugs while minimizing systemic toxicity. Computational modeling can enable rapid evaluation of nanoparticle (NP) designs and numerical optimization. Here, an optimization study was performed using an existing tumor model to find NP size and ligand density that maximize tumoral NP accumulation while minimizing tumor size. Optimal NP avidity lies at lower bound of feasible values, suggesting reduced ligand density to prolong NP circulation. For the given set of tumor parameters, optimal NP diameters were 288 nm to maximize NP accumulation and 334 nm to minimize tumor diameter, leading to uniform NP distribution and adequate drug load. Results further show higher dependence of NP biodistribution on the NP design than on tumor morphological parameters. A parametric study with respect to drug potency was performed. The lower the potency of the drug, the bigger the difference is between the maximizer of NP accumulation and the minimizer of tumor size, indicating the existence of a specific drug potency that minimizes the differential between the two optimal solutions. This study shows the feasibility of applying optimization to NP designs to achieve efficacious cancer nanotherapy, and offers a first step towards a quantitative tool to support clinical decision making.

## Introduction

Targeted cancer nanotherapy relies on nanocarriers to deliver anticancer agents safely to tumors while minimizing systemic toxicity. Nanocarrier-mediated drug delivery has been associated with up to an 8-fold increase in drug efficacy compared to conventional chemotherapy^1^. Both drug and nanocarrier design play important roles in treatment efficacy. In general, drug design focuses on finding compounds that inhibit cancerous cell viability or proliferation, while nanocarrier design aims at developing nano-vehicle structures that maximize drug concentration in tumor relative to healthy tissue, thus reducing adverse drug effects. Experimental and computational methods have been employed to pursue such designs.

Specifically for vasculature-bound nanoparticles, *in vitro* studies have focused on characterizing the effect of nanocarrier design on margination, adhesion, and uptake while flowing through the tumoral vasculature. The tendency for vascular-borne liposomes and metal nanoparticles to drift from the blood streamlines towards the tumor vessel walls was studied by Toy *et al*.^2^ using an *in vitro* microcirculation model. Considering different designs that vary in nanocarrier diameter {56, 60, 65, 100, 300 nm} as well as aspect ratio {0.45, 1}, it was shown that small eccentric nanoparticles are associated with stronger margination tendency. Larger nanoparticles, however, have exhibited a different correlation. Charoenphol *et al*.^3^ examined the margination of nanoparticles of larger diameters {200, 500, 2000, 5000 nm}, flowing in an *in vitro* parallel flow chamber, showing that the margination rate increases with size. Patil *et al*.^4^ measured *in vitro* the adherence strength of nanoparticles with diameters {5, 10, 15, 20 *μ*m} coated with P-selectin glycoprotein ligands, showing that large nanoparticles have strong adherence properties due to a high contact area with the vascular endothelium. Importantly, large nanoparticles are subjected to stronger hemodynamic forces and torques that may dissociate them from vessel walls^5^. Boso *et al*.^6^ measured the accumulation of nanoparticles of {0.75, 1, 2, 4, 6 *μ*m} diameters in a parallel flow chamber. Data was fitted using an artificial neural network to correlate nanoparticle accumulation and size. As the size increases, the accumulation increases until it saturates or starts declining after a certain diameter that ranges between 4 and 6 *μ*m depending on the wall shear rate. The study indicates the presence of moderate nanoparticle size that maximizes the adherence properties.

In addition to the aforementioned *in vitro* studies, *in vivo* investigations have evaluated the overall efficacy of nanoparticles in living subjects. Rostami *et al*.^7^ showed that encapsulating Doxorubicin (DOX) in H6-equipped nanocarriers trebles the inhibition of mammary gland tumors in a mouse model compared to free DOX. Docetaxel-loaded nanoparticles of 349 nm diameter were delivered to mouse mammary tumors in^8^. Significant improvement in antitumor activity was obtained by delivering the drug through nanoparticles. The effect of nanoparticle size was studied by Joshi *et al*.^9^. Liposomes of diameters {60, 80, 200, 650, 670 nm} delivered to gliomas have indicated that 200 nm had the highest uptake rate. Other *in vivo* studies are reviewed in Zhang *et al*.^10^, for which nanoparticle design recommendations were based on increasing circulation time, taking advantage of the enhanced penetration and retention (EPR) effect, and maintaining high drug entrapment efficiency in the nanoparticle synthesis stage.

Quantifying nanotherapy efficacy using *in vitro* assays may require lengthy preparatory steps, which include setting up proper cell lines and reagents, synthesizing nanoparticles, and tailoring experimental protocols. The complexity of these studies is further escalated *in vivo*. The cost and time associated with *in vivo* studies present limitations to evaluating different designs. Not only is acquiring and maintaining animal models expensive, but there may exist a long process from the initiation of oncogenic mutation or transplantation of xenografts, tumor proliferation, to monitoring tumor regression after nanoparticle injection. This also requires advanced imaging techniques and multidisciplinary expertise. For these reasons, computational modeling offers an attractive option for exploratory evaluation of nanoparticle design that complements experimental work, including investigation of a wide range of variables.

Computational modeling of tumor growth and nanoparticle delivery is, however, non-trivial. Such models need to consider a variety of biological processes such as angiogenesis and drug cellular uptake in order to yield informative results. The models typically consist of submodels, coupled sequentially or iteratively, which may be difficult to solve and slow to converge to a solution. Decuzzi *et al*.^11^ modeled the margination of nanoparticles by taking into consideration buoyant and hemodynamic forces, as well as van der Waals interactions. The model explored a wide range of sizes, with results showing that diameters between 100 and 400 nm have relatively slower margination rates. Decuzzi and Ferrari^5^ modeled the probability of nanoparticle adhesion at the vasculature wall. Parameters included nanoparticle size, aspect ratio, vessel wall shear stress, receptor-ligands association constant, ligand density, and receptor density. In parallel, tumor growth in two spatial dimensions coupled with neovasculature development was modeled mathematically in^12–15^. In^16^, Frieboes *et al*. integrated the nanoparticle delivery model of^5^ with the tumor mechanics models of^14,15^ to create a comprehensive model to predict the intra-tumoral distribution and accumulation of vasculature-bound nanoparticles. However, the computational cost of the integrated model hinders the evaluation of all possible nanoparticle designs of interest. Nanospheres of {100, 600, 1000} nm diameters were simulated using different values of nanoparticle avidity and tumor conditions. The results showed that large nanoparticles accumulate at higher rates at the tumor periphery, while smaller nanoparticles have lower adherence strength but are distributed more uniformly throughout the tumor tissue. Van de Ven *et al*.^17^ used the model in^15^ to study the effect of drug potency on tumor growth inhibition and to determine the number of nanoparticles needed to reach a half maximal effective concentration IC_50_. Wu *et al*.^18^ used the model in^15^ to study how the tumor interstitial pressure and fluid flow affect nanoparticle transport and distribution. Recently, the model in^16^ was extended to simulate the tumor response to drug release from vasculature-bound nanoparticles^19^. Further information regarding mathematical characterizations of tumor nanotherapy can be found in^20,21^.

Previous computational and experimental work has primarily investigated the performance of nanocarriers using selected values of design variables such as nanoparticle size, aspect ratio, and ligand density. Optimizing nanoparticle design, however, requires design space exploration, which may be impractical to accomplish solely via empirical methods or computational models. Recently, Chamseddine and Kokkolaras^22^ addressed this issue by applying rigorous optimization to the design of nanoparticles in order to maximize the tumoral nanoparticle accumulation and distribution with respect to the nanoparticle physical and chemical properties. The model was static, i.e., considered a single injection and does not update the tumor size and vasculature in response to the treatment. Although a sensitivity analysis was conducted to prove the robustness of the optimal design with 20% change in the average wall shear stress, the proposed design is not guaranteed to remain optimal if the tumor structure changes drastically. In this paper, optimization is applied to a “blackbox” version of the model, presented in^19^, that considers the dynamic changes in the tumor size and vasculature, enabling to obtain a nanoparticle design that is potentially optimal over the course of treatment. This represents a first step toward the goal of developing a clinically-relevant numerical tool to assist nanoparticle design on a patient-specific basis.

## Methods

### Computational Model

**Figure 1:**
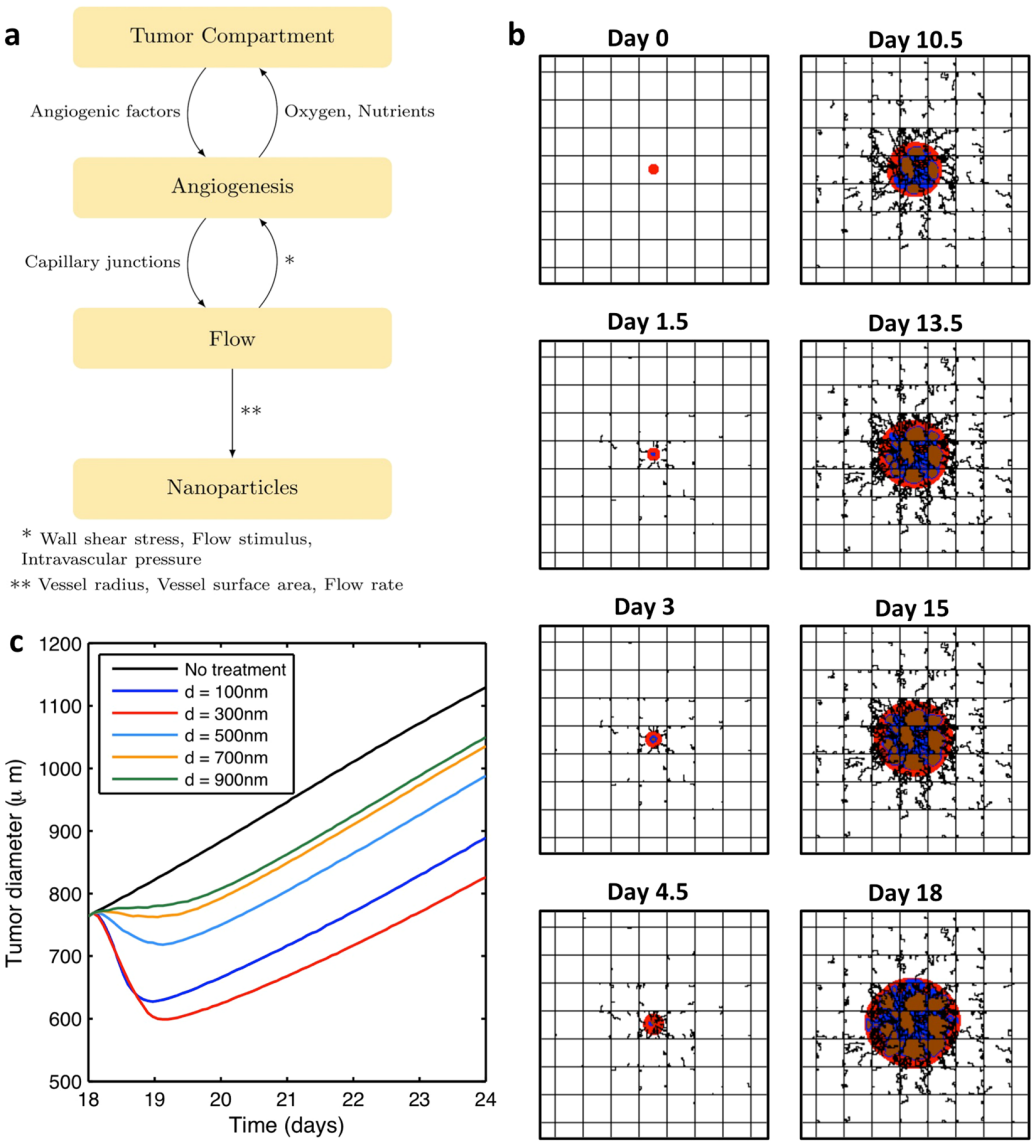
Tumor model used for analysis. (**a**) Interaction among the different submodels as described in^16^. (**b**) Simulated tumor formation due to loss of growth control using the computational model. Viable, necrotic, and hypoxic tissues are represented in red, blue, and brown respectively. Preexisting capillary vessels are represented by the straight orthogonal lines where blood enters from lower left corner to the upper right corner, and flows into the tissue supplying it with oxygen and nutrients. New blood vessels created by the process of angiogenesis are illustrated by irregular lines that sprout from the preexisting vessels. The panel dimensions are 2 × 2 mm. (**c**) Tumor regression in the treatment phase using different nanoparticle diameters. The treatment phase starts after 18 days of tumor growth.

A previously developed numerical model is used to compute tumor growth and response to nanoparticle drug delivery. The model^19^ builds upon the work of^5,13–16^, and is used in this study as a “blackbox” system with a limited set of inputs to calculate nanoparticle accumulation and tumor regression as a function of the nanoparticle design. Briefly, the model is composed of 4 submodels that are coupled in the configuration shown in Fig. 1a^16^. The “Tumor Compartment” submodel computes the progression/regression of the tumor as a function of drug, oxygen, and nutrient concentrations. It also creates hypoxic and necrotic regions, which induce angiogenic factors (TAF). TAF drives the “Angiogenesis” submodel to develop new blood vessels. “Angiogenesis” is coupled with the “Flow” submodel, which determines the wall shear stress among other flow properties in the preexisting and neo-vasculatures. The “Nanoparticle” submodel determines the nanoparticle accumulation and drug release to the cancerous tissue as a function of the nanoparticle design and tumor parameters. The model does not currently include an immune system response. Thus, the nanoparticles are essentially considered hidden from immunosurveillance. Table 1 lists the input parameters to the “blackbox” system and their values as used in this study, unless it is otherwise specified in the text. The complete list of the computational model parameters is reported in^14–16,19^.

**Table 1:** Main parameters used in the computational model.

Parameter	Value
Drug decay rate	4.1588 *s*^−1^
Drug diffusion coefficient	3.334 × 10^−3^ *mm*^2^/*s*
Drug effect	1 (calibrated to drug of moderate potency)
Measure of nanoparticle dissociation tendency (*β* in^16^)	6.63 × 10^−4^ *m*^−2^.*s*
Measure of receptor deficiency (*γ* in^16^)	1.07 × 10^3^ *m*^−1.57^
Nanoparticle avidity (*α* in^16^)	2.95 × 10^10^ *m*^−2^

#### Growth Phase

At day 0, a transformed group of cells is placed in the middle of a two-dimensional panel representing a tissue with blood vessels that are laid orthogonally as shown in Fig. 1b to simulate the regular vascularization of normal tissue. Blood enters the tissue from the bottom left corner to supply oxygen and nutrients. This enables the cancerous cells to proliferate and develop into a mass (tumor) due to the suppression of apoptosis. As the tumor grows, some cancerous cells distal from the blood vessels become hypoxic. This tissue produces TAF to stimulate new blood vessels that sprout from the existing vessels in order to supply the tumor tissue with blood. The neovasculature has an irregular structure and promotes tumor progression as shown in Fig. 1b. The growth phase is stopped at day 18 when the tumor reaches a diameter of 780 *μm*, after which the treatment phase starts.

#### Treatment Phase

Drug-carrying nanoparticles are injected into the blood vessel inlets at day 18. A fraction of the nanoparticles adhere to the tumor vessels. This fraction depends on the nanoparticle design. Anticancer agents are then released to the cancerous tissue. If the drug concentration exceeds a specific threshold, the tissue will die via apoptosis depending on the drug potency (see Table 1).

A preliminary investigation of a 6-day treatment phase was simulated using different nanoparticle diameters. The change in tumor diameter in response is depicted in Fig. 1c. Since the curves do not intersect after 1 day of treatment, the treatment duration does not affect the relative performance for different nanoparticle sizes. Therefore, in our search for the optimal diameter, the treatment phase is stopped after 36 hours of nanoparticle injection, saving substantial computational time. Note that after a certain time of nanoparticle injection, a relapse is observed. This regrowth could be caused by two factors: either the drug is exhausted or the drug does not reach cytotoxic concentration for all of the proliferating tumor tissue.

### Optimization

The ultimate goal of drug-based cancer treatments is to eradicate tumors completely or reduce their size prior to radical treatment intervention as a neoadjuvant therapy. Additionally, the aim of using nanoparticles as drug carriers is to reduce the side-effects associated with conventional chemotherapy while maximizing the drug delivery to the tumor tissue. Hence, we consider two objectives: minimizing the tumor size at the end of the treatment phase, and minimizing the treatment toxicity by maximizing the accumulation of nanoparticles in the tumor. Accordingly, two objective functions are defined:

1. Tumor Diameter (TD): the tumor diameter at the end of the treatment (day 19.5) normalized to its value at the beginning of the treatment (day 18).
2. Tumor Nanoparticles (TNP): the fraction of the injected nanoparticles that adhere to the tumor at the injection time (day 18).

Let **x** denote the set of nanoparticle design variables. In general, the vector **x** may include nanopaticle diameter, aspect ratio, elasticity, ligand density, ligand-receptor affinity constant, drug release rate, and drug load. In this study, we consider spherical nanoparticles (aspect ratio of 1) because they are easy to manufacture^23^, and because they are predominant in current clinical and experimental studies^24,25^. For instance, the clinically proven nanodrug Doxil - used to treat different types of cancers such as breast and ovarian - is composed of 100 nm spheres. The optimal values of *x* that minimize TD, called minimizers of TD, can be obtained by solving:

1 $$\begin{array}{cc}\mathop{min}\limits_{{\rm{x}}\in {{\mathbb{R}}}^{n}} & {\rm{T}}{\rm{D}}({\bf{x}})\\ {\rm{s}}{\rm{u}}{\rm{b}}{\rm{j}}{\rm{e}}{\rm{c}}{\rm{t}}\,{\rm{t}}{\rm{o}} & {{\bf{l}}}_{b}\le {\bf{x}}\le {{\bf{u}}}_{b}\\ {\rm{w}}{\rm{h}}{\rm{e}}{\rm{r}}{\rm{e}} & {{\bf{l}}}_{b},{{\bf{u}}}_{b}\in {{\mathbb{R}}}^{n},\end{array}$$

where *n* is the number of variables considered, and **l**_*b*_ and **u**_*b*_ are the lower and upper bounds that define the feasible region of **x**. Constraints are implemented implicitly in the analysis model. The maximizers of TNP are determined by solving:

2 $$\begin{array}{cc}\mathop{max}\limits_{{\rm{x}}\in {{\mathbb{R}}}^{n}} & {\rm{T}}{\rm{N}}{\rm{P}}({\bf{x}})\\ {\rm{s}}{\rm{u}}{\rm{b}}{\rm{j}}{\rm{e}}{\rm{c}}{\rm{t}}\,{\rm{t}}{\rm{o}} & {{\bf{l}}}_{b}\le {\bf{x}}\le {{\bf{u}}}_{b}\\ {\rm{w}}{\rm{h}}{\rm{e}}{\rm{r}}{\rm{e}} & {{\bf{l}}}_{b},{{\bf{u}}}_{b}\in {{\mathbb{R}}}^{n}.\end{array}$$

Problems (1) and (2) are solved using the Mesh Adaptive Direct Search algorithm (MADS)^26^. Since the gradients of the computational model cannot be approximated reliably, we use MADS, which is a derivative-free optimization algorithm that has rigorous convergence properties. Moreover, the computational model used here as a blackbox for analysis is computationally expensive. To examine a single nanoparticle design, an Intel(R) Core(TM) i7-3770 CPU @ 3.4 GHz processor requires 1.5 hours of CPU time. For this reason, we use a surrogate-assisted optimization approach to reduce the number of computational model evaluations required to obtain the optimal design. Specifically, we utilize the search step of the iterative mesh adaptive direct search (MADS) algorithm^27^ to solve a surrogate of the optimization problem, i.e., we solve the optimization problem using surrogate models of the objective functions. To enhance the efficiency of the MADS algorithm we build and update ensembles of surrogates and use a novel order-based error metric tailored specifically for surrogate optimization to utilize the best surrogate at each iteration^28^. In this manner, we generate several candidates for the next iterate, which we combine with the candidates generated at the poll step of MADS, which is the foundation of its convergence properties. The computational model is then used only to evaluate all these candidates opportunistically to select the next iterate. In other words, we generate a lot of useful information by means of computationally inexpensive surrogate models but make algorithmic decisions using the high-fidelity computational model.

## Results

### Optimizing Nanoparticle Diameter

The drug biodistribution depends on the diameter *d* of the drug-carrying nanoparticles, which are localized depending on their size^16,22,29^. Small nanoparticles are associated with longer circulation time, increasing their chance to reach the tumor. Their adherence properties are poor, however, due to the low contact area between ligands on the surface of the nanoparticles and receptors over-expressed in the vascular endothelium of the malignant lesion. On the other hand, large nanoparticles have strong binding affinity, but they are also exposed to high hemodynamic loadings that may dissociate them from the endothelium. In addition, large nanoparticles tend to accumulate at the periphery of the tumor or bind to healthy tissues before they reach the tumor site due to their low circulation time. The optimal nanoparticle diameter d^*^ lies within this range and is obtained by solving Problems (1) and (2) while setting **x** = [*d*]. Nanoparticles smaller than 10 nm are exposed to renal clearance, and nanoparticles larger than 1000 nm may not be able to flow in narrow tumor vessels and cause embolism. Therefore, *d* lies between **l**_*b*_ = 10 nm and **u**_*b*_ = 1000 nm in this study. For this evaluation, the nanoparticle avidity *α* = 2.95 × 10^10^ *m*^−2^, calculated by considering typical values for the receptor density, ligand density, and receptor-ligand binding constant under zero load, which are in the order of 10^12^#/*m*^2^, 10^14^#/*m*^2^, and 10^−14^ *m*^2^ respectively^16^.

**Figure 2:**
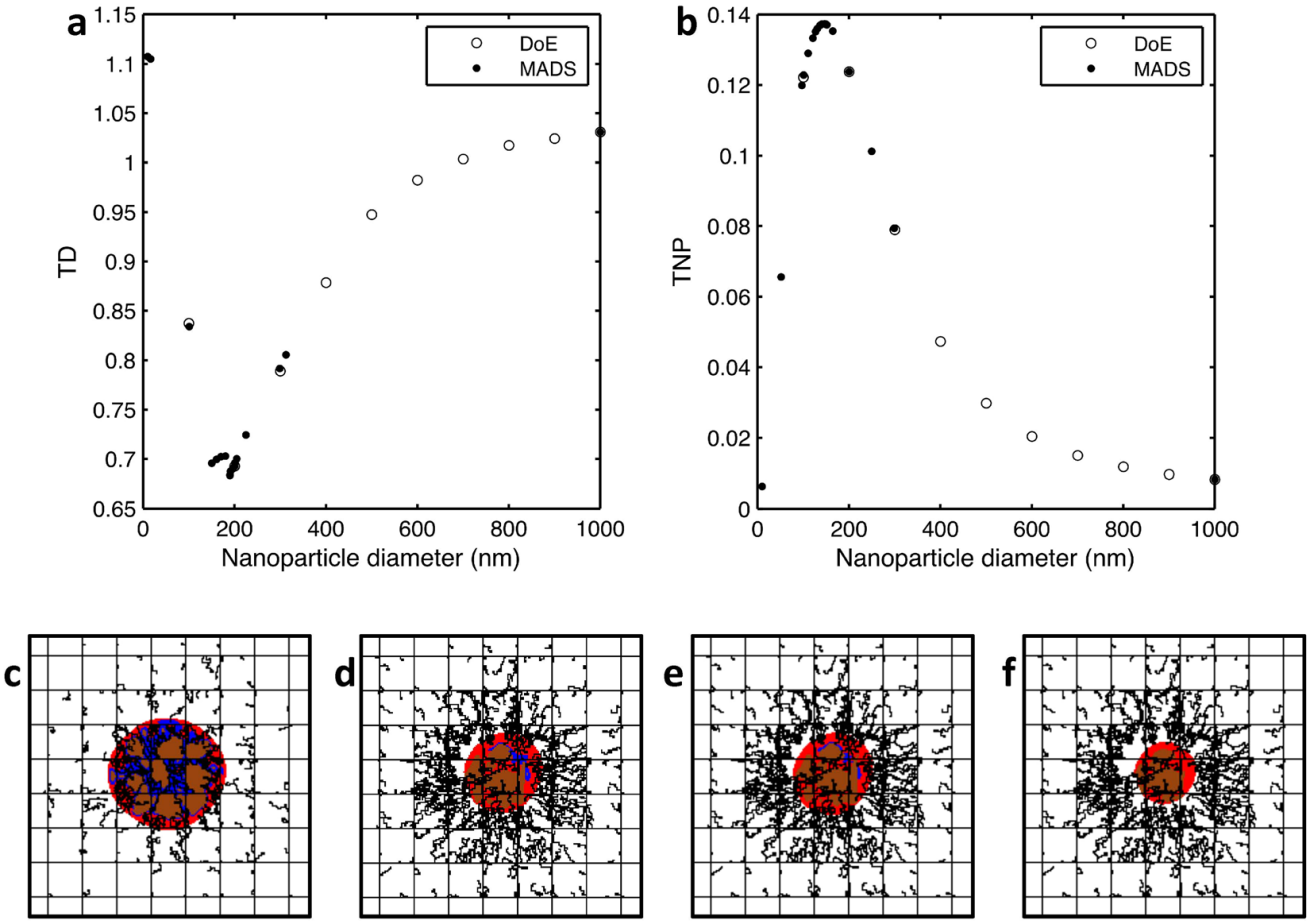
Simulation of tumor treatment using optimal nanoparticle designs. Comparison of MADS-selected points with sample designs selected manually to find the (**a**) minimizer of TD, and (**b**) maximizer of TNP, where TD the tumor diameter at the end of the treatment normalized to that at the beginning of the treatment and TNP is the fraction of the injected nanoparticles that accumulate at the tumor. Simulated tumor (**c**) before treatment, (**d**) after 36 hours of treatment using the minimizer of TD d* = 190 nm showing a reduction to 68.3% of its initial diameter, (**e**) after 36 hours of treatment using the maximizers of TNP d* = 147 nm showing a reduction to 69.6% of its initial diameter, and (**f**) after 36 hours of treatment with nanoparticles of $({d}^{\ast }=334\,nm,{\alpha }^{\ast }=1\times 10\,{m}^{-2})$ obtained after expanding **x** to [*d*, *α*], showing a reduction to 50.5% of its initial diameter. Panel dimensions are 2 × 2 mm.

Figure 2a compares empirically-selected points with MADS-selected points in an attempt to minimize TD. In empirical methods, trial points are randomly chosen; however, using MADS, trial points are selected systematically to converge to the optimal solution. Note that when MADS approaches the optimal diameter, it tries many points in the vicinity before terminating at the best solution. The obtained optimal nanoparticle diameter up to 1 nm accuracy is d^*^ = 190 nm, reducing TD^*^ = 0.683.

Similarly, problem (2) is solved to maximize the tumor nanoparticle accumulation. The progress of MADS is shown in Fig. 2b. The optimal nanoparticle size that maximizes TNP is d^*^ = 147 nm leading to TNP^*^ = 0.137; i.e., around 14% of the injected nanoparticles successfully reach and adhere to the tumor. The corresponding TD is 0.484. Both optimal solutions are summarized in Table 2. Figure 2d,e shows the regression of the tumor by injecting both optimal solutions.

**Table 2:** Solutions of Problems (1) and (2) with **x** = [*d*].

	Objective Function	Optimizer	Optimum
*Minimize*	Tumor Diameter (normalized)	d^*^ = 190 nm	TD^*^ = 0.683
*Maximize*	Tumor Nanoparticle Fraction	d^*^ = 147 nm	TNP^*^ = 0.137

### Effect of Nanoparticle Avidity

Vasculature-bound nanoparticles are equipped with ligands of high binding affinity to receptors over expressed in the vascular endothelium of tumor vessels. In the model, we assume that the integrin *α*_*ν*_*β*_3_ exists with an area density *m*_*r*_ in the malignant lesion. Corresponding ligands such as vitronectin^30^, fibronectin^31^, fibrinogen^32^, and osteopontin^33^ are available at the surface of the nanoparticles with density *m*_*l*_. Each receptor-ligand pair has a certain affinity that is quantified by the binding constant under zero load ${K}_{A}^{0}$. The nanoparticle avidity $\alpha \propto {m}_{r}{m}_{\ell }{K}_{A}^{0}$ corresponds to the overall affinity of the nanoparticle^16^.

The optimization problems (1) and (2) are solved again using a different value of *α* to check if the optimal diameters change. Changing the parameter *α* has an effect on the optimal nanoparticle diameters as shown in Table 3, where the cases of *α* = 1 × 10^11^ *m*^−2^ and *α* = 1 × 10^12^ *m*^−2^ are listed. It can be observed that as *α* increases, the diameter that maximizes TNP decreases. This decrease in d^*^ can be explained by the increased ligand and receptor densities to the point that the nanoparticle-endothelium contact area required to cause binding is reduced. The minimal TD is altered, however, since smaller nanoparticles have lower drug loads. Since the value *α* has an effect on the optimal nanoparticle diameter, it is necessary to add it to the set of variables to optimize it along with *d* in an all-in-one optimization problem.

**Table 3:** Solutions of Problems (1) and (2) with **x** = [*d*] using different values of *α*.

Solution of	Problem (1)		Problem (2)	
*α* = 2.95 × 10^10^ *m*^−2^	d^*^ = 190 nm	TD^*^ = 0.683	d^*^ = 147 nm	TNP^*^ = 0.137
*α* = 1 × 10^11^ *m*^−2^	d^*^ = 90 nm	TD^*^ = 0.866	d^*^ = 69 nm	TNP^*^ = 0.137
*α* = 1 × 10^12^ *m*^−2^	d^*^ = 230 nm	TD^*^ = 1.08	d^*^ = 15 nm	TNP^*^ = 0.137

#### Treating Nanoparticle Avidity as a Design Variable

Let **x** = [*d*, *α*]^*T*^. The range of *α* is assumed to be between 10^10^ and 10^12^ *m*^−2^ complying with typical ranges of *m*_*r*_, *m*_*l*_, and ${K}_{A}^{0}$^5,16^. Hence, the feasible design space is l_*b*_ = [10 *nm*, 10^10^ *m*^−2^]^*T*^ and u_*b*_ = [1000 *nm*, 10^12^ *m*^−2^]^*T*^. Results for minimizing the tumor diameter and maximizing the tumor nanoparticle accumulation are shown in Table 4.

**Table 4:** Solutions of Problems (1) and (2) with **x** = [*d*, *α*]^*T*^.

	Objective Function	Optimizer	Optimum
*Minimize*	Tumor Diameter (normalized)	$[{d}^{\ast }=334\,nm,{\alpha }^{\ast }={10}^{10}\,{m}^{-2}]$	TD^*^ = 0.505
*Maximize*	Tumor Nanoparticle Fraction	$[{d}^{\ast }=288\,nm,{\alpha }^{\ast }={10}^{10}\,{m}^{-2}]$	TNP^*^ = 0.137

Optimizing both nanoparticle diameter and avidity provides a better tumor reduction. Figure 2f displays the tumor at the beginning and end of the treatment, showing that the tumor reduces to 50.5% of its diameter at the start of treatment. Note that the optimal value of *α* lies at its lower bound (10^10^ *m*^−2^) for both problems. The corresponding optimal diameter is increased to maintain an adequate contact area with the endothelium.

#### Relaxing the *α*–Boundary Constraint

**Figure 3:**
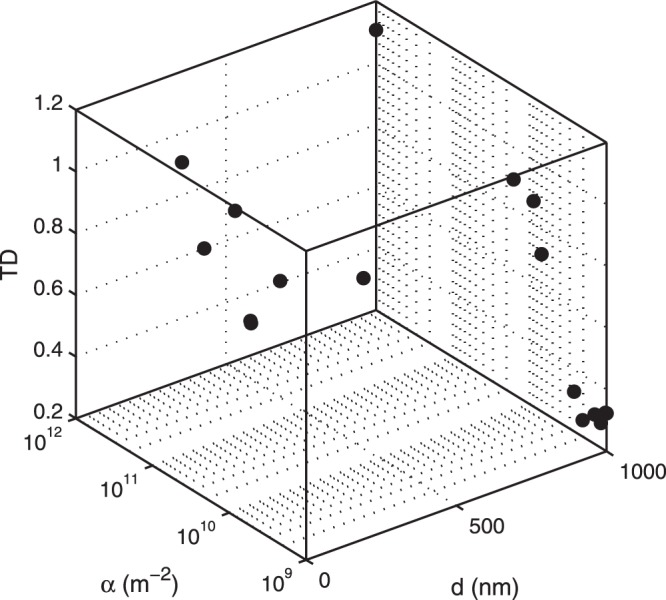
The blackbox evaluation points showing that the design space has been sampled adequately with convergence to the optimal values of *d* and *α* that minimize TD.

The lower bound of *α* is an active bound; i.e., if it changes, the optimal value of *α* changes. To examine the proposed nanoparticle design practice of reducing the value of *α* and selecting a proper diameter, we reduce the lower bound of *α* to 10^9^ *m*^−2^ and check if α^*^ remains a boundary optimum. Figure 3 plots MADS progression toward the optimal solution of the relaxed problem. The solution confirms the existence of α^*^ at the active bound. The variable d^*^ remains an interior optimum having a value of 980 nm, increased to compensate for low ligand density.

### Robustness of the Optimal Design

The solution for problems (1) and (2) may change if the tumor morphology changes. Although numerical optimization can be a powerful tool that supports precision medicine dealing with patient-specific situations, designs that are aimed at treating a wide range of patients need to be insensitive to changes in tumor parameters. A rigorous method to attain robust designs is to optimize under uncertainty^34^, which will be addressed in future work. Here, we perform a sensitivity analysis with respect to model parameters that characterize the tumor microenvironment. We identify *β* and *γ* as candidates for altering the optimal design. The parameter *β* ∝ *χμ*/(*k*_*B*_*Tm*_*r*_) combines Boltzmann thermal energy, blood viscosity, ligand-receptor binding force, and receptor density. The parameter *γ* is inversely proportional to the receptor density. More details about these parameters can be found in^5,16^.

Let *Z*_*d*_, *Z*_*α*_, *Z*_*β*_, *Z*_*γ*_ be categorical variables that measure *d*, *α*, *β*, and *γ* respectively. Each categorical variable has a value that belongs to the set {1, 2, 3} referring to {Low, Medium, High}. For example, the set **Z**_*d*_ = {1, 2, 3} refers to **d** = {10, 500, 1000}*nm*. Similarly, **Z**_*α*_ = {1, 2, 3} means *α* = {1*e*10, 1*e*11, 1*e*12} *m*^−2^. Considering 3 levels for four variables, there exist 81 permutations of the vector [*Z*_*d*_, *Z*_*α*_, *Z*_*β*_, *Z*_*γ*_]^*T*^.

**Figure 4:**
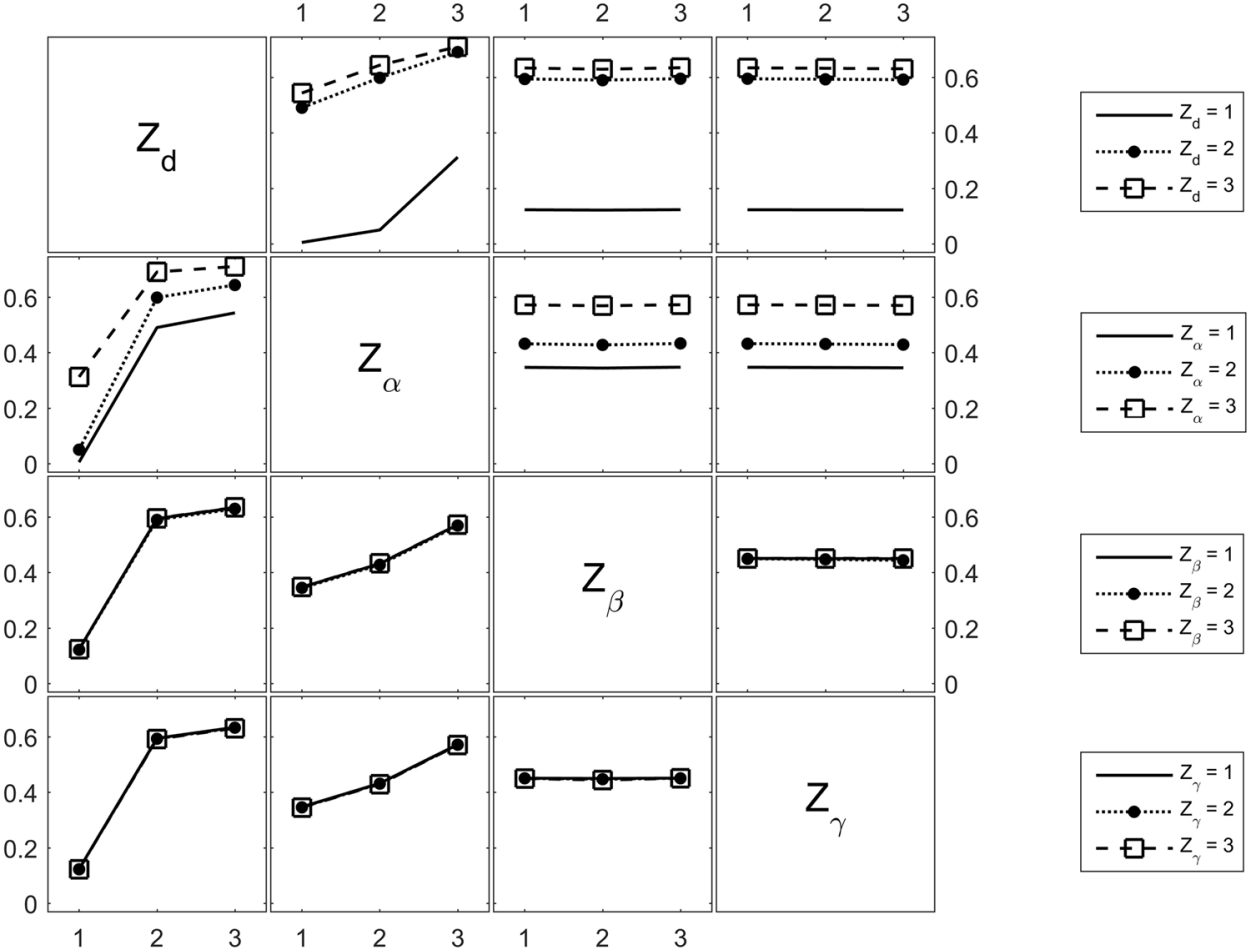
Interaction plot of *d*, *α*, *β*, and *γ* with the output TNP. The 4 categorical variables referring to the parameters *d*, *α*, *β*, and *γ* are represented in the diagonal plots. The horizontal axis of each off-diagonal plot is the categorical variable on the diagonal vertically above the plot (if the plot is in a lower triangular position) or below the plot (if the plot is in an upper triangular position). The vertical axis of each off-diagonal plot is the tumor nanoparticle accumulation TNP.

The parameters *β* and *γ* are expected to have a direct impact on nanoparticle accumulation because they model nanoparticle-to-endothelium interactions. Therefore, we investigate the change in TNP with respect to the input vector using the interaction plot of Fig. 4. The interaction plot is a matrix plot, where the diagonal of the plot displays the categorical variables. The interaction of the parameter highlighted at the row-diagonal (i, i) with the parameter at the column-diagonal (j, j) is displayed at the off-diagonal position (i, j). For instance, the subplot (1, 2) plots the interaction of *d* and *α*. The horizontal axis is *Z*_*α*_, the vertical axis is the output TNP, and the different lines are the different values of *Z*_*d*_ (indicated on the legend to the right of the corresponding row). The contribution of *β* and *γ* is illustrated in the subplots (1, 3), (1, 4), (2, 3), (2, 4), (3, 1), (3, 2), (3, 4), (4, 1), (4, 2), and (4, 3). In all of these plots, the graphs are either horizontal or coincide. Therefore, nanoparticle accumulation highly depends on the nanoparticle design and less on tumor biological conditions such as receptor density and blood properties.

### Effect of Drug Potency

The potency of the drug encapsulated in the nanoparticles has an important role in tumor regression^17^. Drugs with higher potency cause fast shrinkage but are associated with high systemic toxicity. On the other hand, drugs with lower potency may evince slower tumor regression but have higher median toxic dose, lowering the associated adverse events. The computational model accounts for the drug potency through the proliferative term ${\lambda }_{p}=[\sigma \mathrm{(1}-{\bar{\lambda }}_{effect}{\bar{C}}_{D}{{\bf{1}}}_{D > {T}_{drug}})-A]$, which quantifies the interplay between cell mitosis, promoted by the availability of nutrients and oxygen *σ*, and cell apoptosis, which occurs if the drug concentration *D* exceeds a specific threshold *T*_*drug*_ in the tissue^17,19^. The drug potency is measured by ${\bar{\lambda }}_{effect}$ having a unit of effect per drug concentration. Clinically, drugs with higher potency have lower half maximal inhibitory concentration (IC_50_). The parameter *C*_*D*_ is a rescaling factor and *A* is the natural apoptosis rate.

In the Effect of Nanoparticle Avidity section, a hypothetical drug of moderate potency was used. The parameter ${\bar{\lambda }}_{effect}$ was normalized to 1 for the drug considered. Drugs with higher potency are characterized by ${\bar{\lambda }}_{effect}$ > 1, while drugs have a value of ${\bar{\lambda }}_{effect}$ between 0 and 1. Although drug potency does not affect nanoparticle accumulation, it has an impact on the amount of drug needed to induce cell apoptosis, which is expected to change the minimizers of TD.

**Figure 5:**
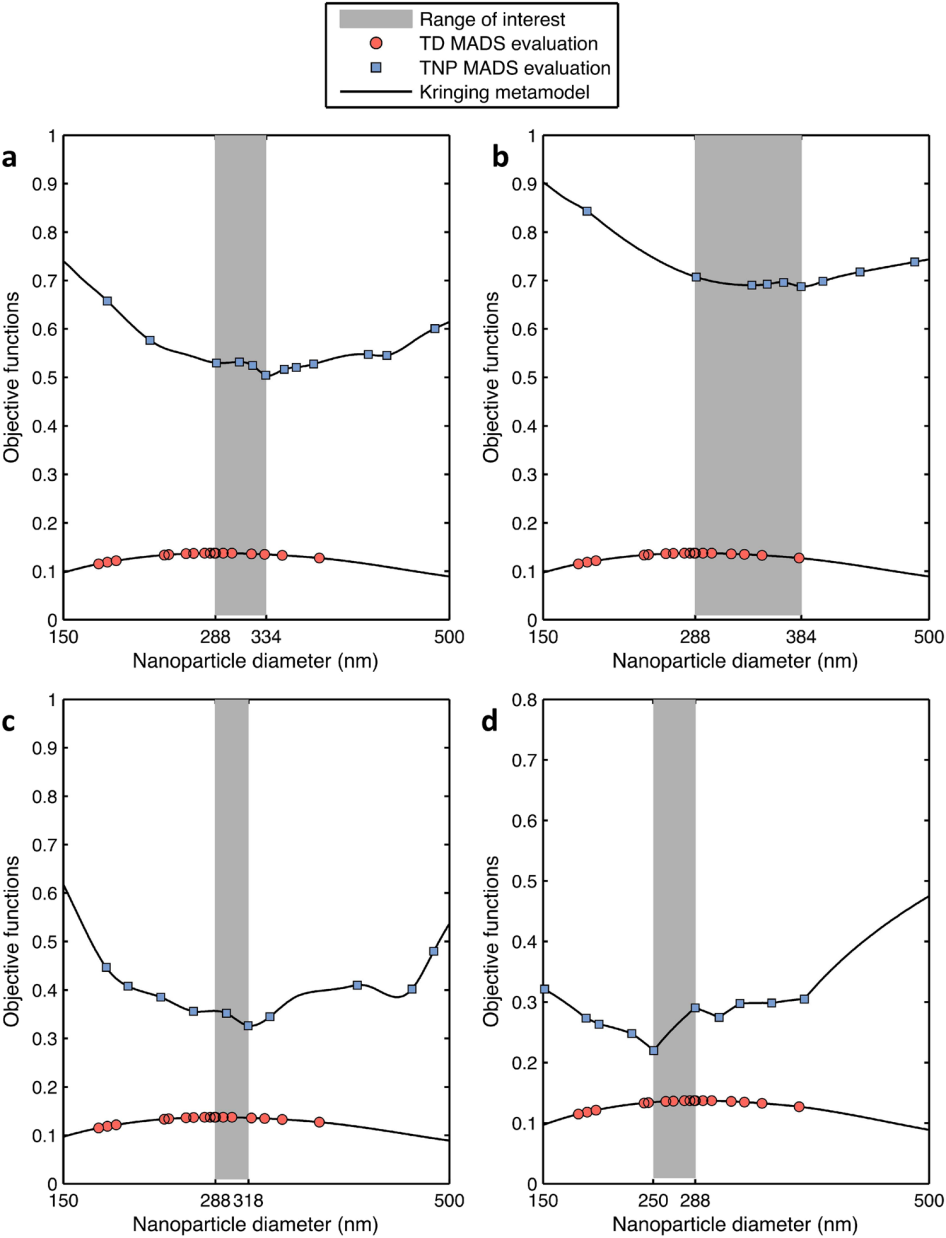
Effect of drug potency on the optimal nanoparticle design. Optimal nanoparticle diameters and local variations at *α* = 1*e*10 *m*^−2^ and (**a**) ${\bar{\lambda }}_{effect}$ = 1 (Doxorobicin), (**b**) ${\bar{\lambda }}_{effect}$ = 0.5 (Ponatinib), (**c**) ${\bar{\lambda }}_{effect}$ = 2 (Lestaurtinib), and (**d**) ${\bar{\lambda }}_{effect}$ = 5 (Midostaurin).

We showed before that there is a difference between the optimal diameter ${d}_{{\rm{TD}}}^{\ast }$ that minimizes TD and ${d}_{{\rm{TNP}}}^{\ast }$ that maximizes TNP. Figure 5a plots both optimal diameters for the case of *α* = 1 × 10^10^ *m*^−2^. If nanoparticles are smaller than ${d}_{{\rm{TD}}}^{\ast }=334\,nm$, less drug is released to the tissue and thus TD is higher. If nanoparticles are larger than ${d}_{{\rm{TD}}}^{\ast }=334\,nm$, they aggregate toward the tumor margin, reducing the tumor diameter exposed to the drug. If nanoparticles are smaller than ${d}_{{\rm{TNP}}}^{\ast }=288\,nm$, their probability to adhere to the tumor site is lower due to the small contact area between the nanoparticle and tumor vessel wall. If nanoparticles are larger than ${d}_{{\rm{TNP}}}^{\ast }=288\,nm$, they are exposed to higher dissociative hemodynamic loadings that return them to the bloodstream. Between the two optimal solutions ${d}_{{\rm{TNP}}}^{\ast }=288\,nm$ and ${d}_{{\rm{TNP}}}^{\ast }=334\,nm$, there exists a region where the treatment shows the most favorable outcome. In this desired region, the nanoparticle design can be selected depending on the weight given to each treatment attribute - lower toxicity versus faster treatment.

#### Parametric Study with respect to Drug Potency

Figure 5 implies that the maximal therapeutic potential is not necessarily tied to the maximal nanoparticle accumulation. In fact, depending on drug potency, a smaller number of large nanoparticles can cause better tumor reduction than smaller nanoparticles. This defines the shaded zone of Fig. 5a. The case of a drug with lower potency (${\bar{\lambda }}_{effect}$ = 0.5) is then considered to investigate the change in the ${d}_{{\rm{TD}}}^{\ast }$. For instance, assume a drug with ${\bar{\lambda }}_{effect}$ = 1, used in previous sections, refers to Doxorubicin. If we consider the case of targeting invasive breast carcinoma, for which mutated MDA-MB-231 cell lines are a representative example with Doxorubicin IC_50_ of 1.24 *μM*^35^, then ${\bar{\lambda }}_{effect}$ = 0.5 refers to a drug of IC_50_ = 2.48 *μM*, similar to Ponatinib^35^. The shaded zone becomes wider since ${d}_{{\rm{TD}}}^{\ast }$ is increased to 384 nm to provide higher drug volume needed in the tissue (Fig. 5b). In what follows, we assume ${\bar{\lambda }}_{effect}$ = 1 refers to Doxorubicin and we use it as a benchmark to make all other comparisons with drugs of different values of ${\bar{\lambda }}_{effect}$.

In addition, a drug with higher potency is studied by setting ${\bar{\lambda }}_{effect}$ to 2, simulating Lestaurtinib for example. Figure 5c shows that the width of the shaded region decreases. If ${\bar{\lambda }}_{effect}$ increases further to 5, for instance if Midostaurin is used, the minimizer of TD becomes less than the maximizer of TNP. The reason is that a large drug load per nanoparticle is not needed to cause apoptosis at high values of ${\bar{\lambda }}_{effect}$. Therefore, the optimal solution shifts to small nanoparticles because they distribute more uniformly.

Notably, in all the considered cases, a small sacrifice in TNP leads to an increase in TD. Therefore, from a computational point of view, nanoparticle designs should be driven by minimizing TD. However, this conclusion may not be generalized; it requires extensive experimental support and should be evaluated for specific tumors. Furthermore, the reason TNP is less sensitive to the nanoparticle design could be due to an implicitly specified model parameter. Determining the drug potency that minimizes the discrepancy between the optimizers of the two objective functions would reduce the tradeoff between treatment speed and toxicity.

#### Conjugating ${d}_{{\rm{TD}}}^{\ast }$ and ${d}_{{\rm{TD}}}^{\ast }$

In order to find a single nanoparticle diameter that optimizes both objective functions, we define the optimization problem

3 $$\begin{array}{ll}\mathop{{\rm{\min }}}\limits_{{\bar{\lambda }}_{effect}\in {\mathbb{R}}} & {(d({\bar{\lambda }}_{effect})-argmax({\rm{TNP}}))}^{2}\\ {\rm{subject}}\,{\rm{to}} & 0.2\le {\bar{\lambda }}_{effect}\le 10\\ {\rm{where}} & \begin{array}{c}d=argmin({\mathrm{TD}|}_{{\bar{{\rm{\lambda }}}}_{{\rm{effect}}}})\\ argmax({\rm{TNP}}\mathrm{)=288}\,nm\mathrm{.}\end{array}\end{array}$$

**Figure 6:**
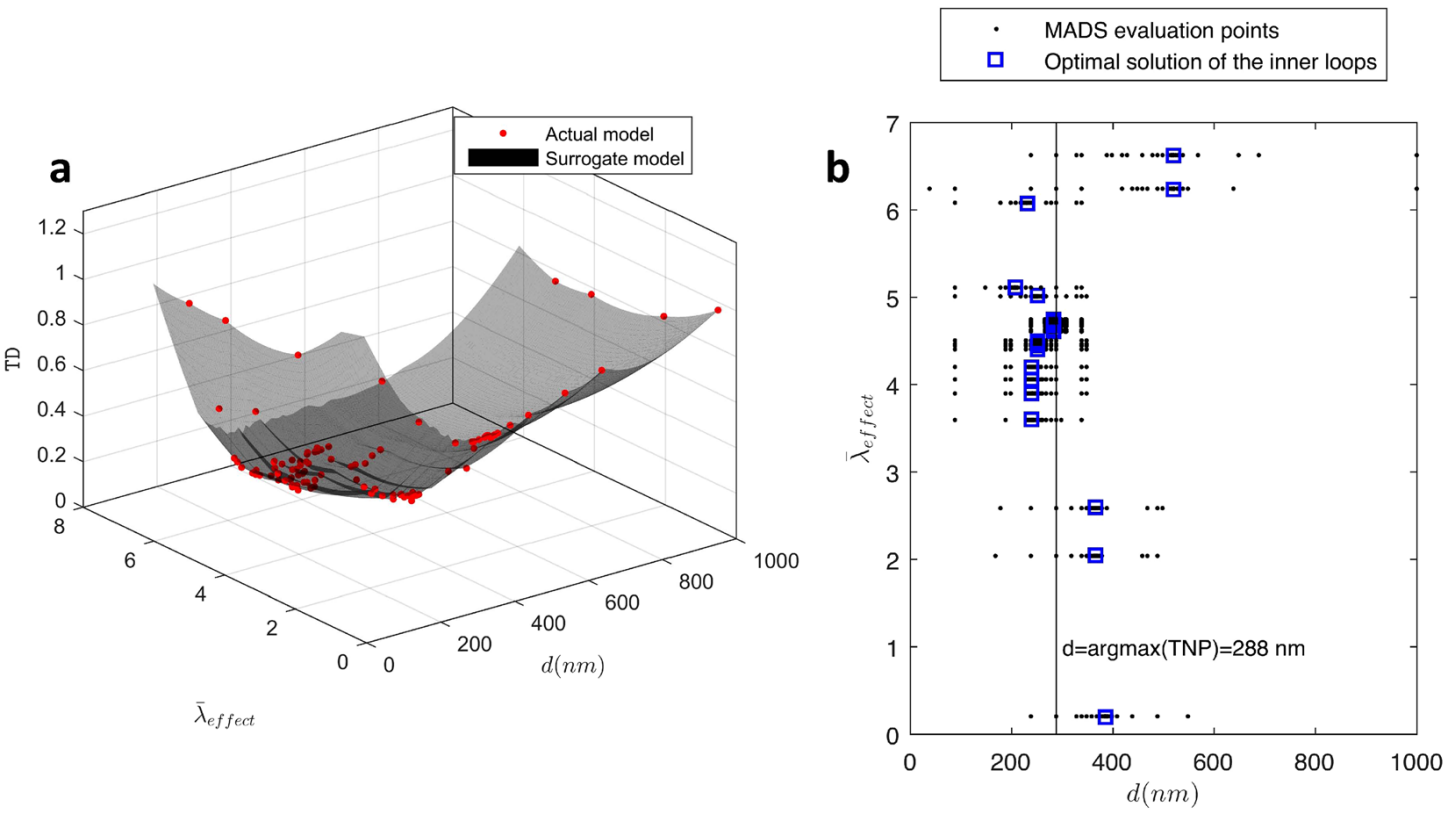
Optimization of drug potency. (**a**) Surrogate of the true model synthesized using kriging method of interpolation. (**b**) Progress of MADS in solving the inner and outer loops of problem (3).

Problem (3) aims at determining the drug potency at which the minimizer of TD coincides with the maximizer of TNP (288 nm). Solving problem (3) requires two loops. The inner loop computes ${d}_{{\rm{TD}}}^{\ast }$ given a drug potency ${\bar{\lambda }}_{effect}$ that is specified by the outer loop. The outer loop iterates to minimize the difference between ${d}_{{\rm{TD}}}^{\ast }$ and 288 nm with respect to ${\bar{\lambda }}_{effect}$. In each outer loop iteration, the inner loop has to complete a full optimization process to find the minimizer of TD. The nested nature of problem (3) requires extensive computational time, which could exceed a month if the computational model used as a blackbox for analysis is used. Alternatively, a surrogate model is created by fitting the points that were evaluated earlier. Figure 6a shows the kriging metamodel constructed using the DACE (Design and Analysis of Computer Experiments)^36^. Exponential correlation functions and second-order polynomial regression models are employed to generate the kriging metamodel.

The solution process of problem (3) is illustrated in Fig. 6b. Each inner loop has a fixed value of ${\bar{\lambda }}_{effect}$ on the vertical axis. Given ${\bar{\lambda }}_{effect}$, MADS visits the surrogate model and finds *d* that is closest to 288 nm, marked by the vertical line in the plot. The inner loop iterates horizontally to converge to the optimal solutions, shown in crosses. Then ${\bar{\lambda }}_{effect}$ changes in the outer loop and the same procedure repeats until the optimal solution ${\bar{\lambda }}_{effect}^{\ast }$ is obtained. The minimal difference between ${d}_{{\rm{TD}}}^{\ast }$ and ${d}_{{\rm{TNP}}}^{\ast }$ is 6 nm. It is attained at the optimal drug potency ${\bar{\lambda }}_{effect}^{\ast }=4.6$, a property similar to Omipalisib in treating the MDA-MB-231 cell line, where the minimizer of tumor diameter is 282 nm, which corresponds to 27% of the tumor diameter at the start of treatment.

### Multiple Treatment Cycles

**Figure 7:**
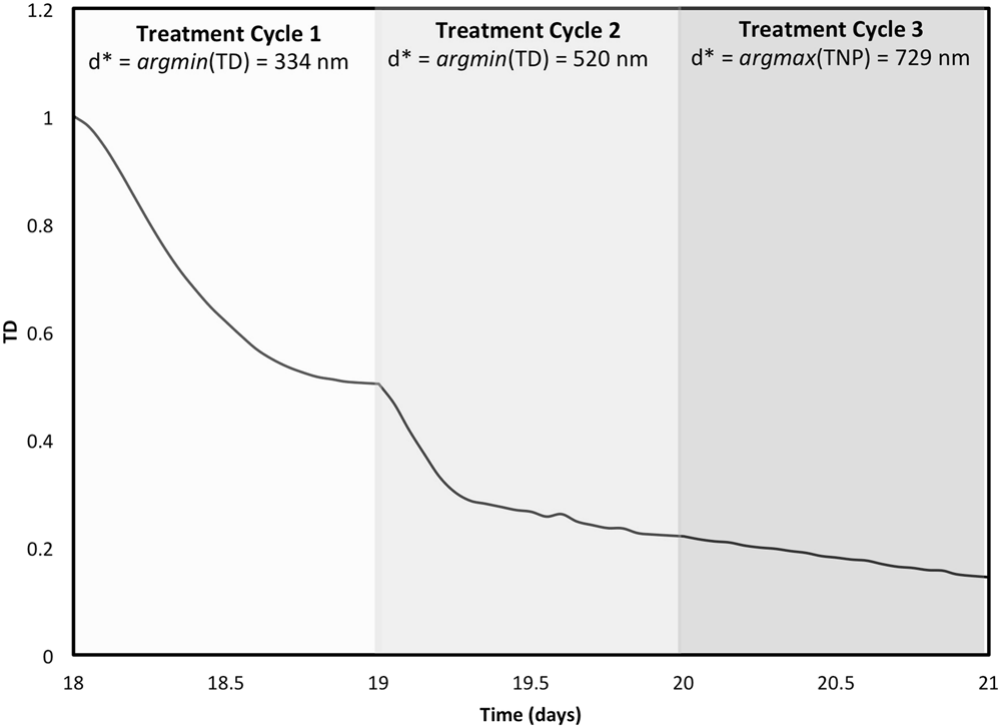
Multiple Injections. The regression of tumor diameter in response to multiple injections of nanoparticles, the sizes of which are optimized before each injection.

We consider the case where ${\bar{\lambda }}_{effect}$ = 1 and *α* = 10^10^ *m*^−2^. A treatment with *d* = 334 *nm* showed a 50% reduction in tumor diameter, illustrated in Fig. 2f. In this section, we simulate three injections to reduce TD further assuming daily administration of nanoparticles. We obtained the optimal nanoparticle diameter for the second cycle of treatment by solving the optimization problem of equation (1) setting **x** = [*d*] to minimize the tumor diameter at the end of the second cycle. By injecting ${d}^{\ast }=520\,nm$, TD is further reduced to 0.22. Becoming very small, the treatment may be maintained in the third cycle using multiple nanoparticle diameters and not merely through the minimizer of TD. For this reason, the maximizer of TNP is chosen for the third injection to economize on the toxicity while achieving the same objective of continuous reduction in TD. The solution of TNP maximization in the third cycle is d* = 729 nm. The change in TD in the three treatment cycles is shown in Fig. 7.

## Discussion

This study applies optimization to the design of drug-carrying nanoparticles targeting tumor vascular-endothelium. Empirical methods to obtain optimal designs are typically based on trial and error schemes. Instead, an optimization approach systematically converges to the optimal design using a significantly lower number of trial points. The reduction in computational time is necessary since optimal solutions are subject to change due to variations in tumor morphology, patient status, and cancer type^37^. Our previous study^22^ was a first attempt to engage rigorous numerical optimization with the design of nanoparticles. We based our analysis on a model that simulates the distribution of nanoparticles and drug release to the tissue without considering the tumor response. In this study, we combined the optimization technique of^22^ with a computational model of tumor nanotherapy^19^ that models tumor evolution and includes complex biological processes such as angiogenesis and tissue necrosis^13–16^. This enables more reliable nanoparticle designs that are optimal for the whole duration of the treatment.

First, the nanoparticle diameter was optimized. Previous studies have predicted that the optimal design moderately lies between lower, more uniform nanoparticle distribution, and higher less uniform distribution^16,19,38^. Experimental studies have examined a finite set of diameters to approximate the ideal size using *in vitro* and *in vivo* studies as reviewed in the Introduction and in^39,40^. Computationally, various diameters were investigated to correlate nanoparticle size and pharmacokinetics^29^. In^16^, three diameters were studied ({100, 600, 1000} nm). The respective tumor accumulation was {12.2%, 2%, 0.8%}. In comparison, the optimal diameter of 147 nm obtained in this study yielded accumulation of 13.7%.

The set of design variables has been expanded to include nanoparticle avidity because it has an impact on nanoparticle distribution^41^. Multidimensional search schemes give rigorous optimization a computational advantage over brute force methods that change one variable at a time. Optimizing nanoparticle diameter and avidity in an all-in-one problem shows a 1.6-fold decrease in tumor diameter and the same percent of nanoparticle accumulation as compared to optimizing the nanoparticle diameter alone. The resulting optimal diameters that maximize tumor targeting and minimize tumor diameter are 288 nm and 344 nm respectively. These diameters lie in the range that benefit from the EPR effect^6^.

The values of the optimal solution have a significant physical meaning. Previous studies have discussed that one way to prolong nanoparticle circulation is size reduction^42–44^. The results suggest that decreasing the nanoparticle avidity is a substitute approach in order to leverage the therapeutic potential of larger nanoparticles (of higher drug load). To further investigate, we reduced the lower bound of the nanoparticle avidity. The results confirm the existence of optimal nanoparticle avidity at the lower bound, whereas the optimal nanoparticle diameter increased to compensate for the decrease in the ligand-receptor pairing per unit area. This conclusion is specific for vasculature-bound nanoparticles where the drug is released at the endothelial layer. For the case where nanoparticles internalize to the tissue, smaller nanoparticles show higher cellular uptake rate, as reported in^45–47^.

In addition, small nanoparticles incur manufacturability limitations in terms of drug load since the efficiency of encapsulating free drugs depends on the nanoparticle size^8,48–55^. For instance, we refer to the preparation of Doxorubicin-loaded albumin nanoparticles in^48^, as Doxorubicin is one of the most widely used antineoplastic agents^56^. The minimum nanoparticle size produced in^48^ is 128 nm and was associated with 58% entrapment efficiency. However, based on our recommendation of using lower ligand density and moderate diameter of 334 nm, the entrapment efficiency could reach up to 78%. Hence, avoiding the unnecessary decrease in nanoparticle size may remove a fundamental constraint that hinders the development of efficient drug-loaded nanocarriers.

The robustness of the optimal design with respect to key tumor properties was then examined by studying the interaction among different parameters (Fig. 4). The results show that nanoparticle accumulation predominantly depends on the nanoparticle design rather than on tumor vessels properties such as receptor density, blood viscosity, and temperature.

The results indicate that maximizing nanoparticle accumulation is not optimal with respect to the tumor diameter reduction. Larger nanoparticles are needed to yield better tumor regression due to the associated high drug load. This was confirmed by running a study that uses a drug of lower potency. Results showed that the minimizer of tumor diameter is larger. Therefore, the optimal design is not unique; however, a range of optimal values can be recommended. This range is bounded by the maximizer of nanoparticle accumulation and minimizers of tumor diameter, where a tradeoff between the two objective functions exist. Depending on the patient status and clinical evaluation, an optimal solution can be selected to satisfy the weight given for each treatment attribution (fast tumor size reduction versus low systemic toxicity). In contrast, when the drug potency is high, less drug load per nanoparticle is required, and the width of the tradeoff range decreases. We increased the drug potency until the minimizer of tumor diameter is smaller than the maximizer of tumor accumulation. If the drug potency is high, smaller nanoparticles are expected to yield better tumor shrinkage because they distribute more evenly. These results are consistent with^16,19^.

The results motivated another study to find the drug potency at which minimizing tumor diameter is tied to maximizing nanoparticle accumulation. If this drug potency exists, nanoparticles could be designed to eliminate anticipated tradeoffs between the desired treatment attributes. Therefore, we formulated an optimization problem to find the drug potency that minimizes the difference between the two optimal designs. The complexity of the optimization problem leads us to synthesize a surrogate model using kriging metamodeling. The solution of the optimization problem indicates that a drug potency that is 4.6 times higher than the original value yields a minimal difference between the two optimal diameter values. A drug of this property would be similar to Omipalisib of IC_50_ = 0.27 *μM*, shown to eliminate MDA-MB-231 cell lines^35^.

Integrating drug potency in the design of nanoparticles enhances drug targeting. Consider for instance the case of invasive breast carcinoma. Choice of targeted drugs such as small molecule inhibitors must be made depending on the driver oncogene responsible for the patient tumor. Talazoparib and Olaparib are two drugs used to target PARP1 and PARP2 genes that could be mutated in advanced breast tumors. Their IC_50_ are 4.86 *μM* and 16.6 *μM* respectively^35^. This corresponds to ${\bar{\lambda }}_{effect}$ of 3.9 for Talazoparib and 13.4 for Olaparib. Potentially, the IC_50_ could be evaluated in the laboratory with the patient’s tumor cells post biopsy, e.g., via monolayer or 3D cell culture cytotoxicity studies in order to help determine which drug is stonger. Toxicologically, Talazoparib represents the better option to target PARP1 and PARP2. If, however, the nanoparticle design that minimizes tumor diameter is predicted by the model to lead to low nanoparticle accumulation, i.e., wide grey region of Fig. 5, then minimizing TD with nanoparticles loaded with Olaparib may offer a better choice with nanoparticle diameters that are closer to the maximizer of TNP.

We further used the optimization approach repetitively to explore the optimization of multiple treatment cycles. At the beginning of each cycle, we obtained the nanoparticle diameter that minimizes the tumor diameter before the next cycle starts. As the tumor size reduces, it became possible for multiple nanoparticle diameters to induce antitumor activity close to that of the minimizer of tumor diameter. For this reason, we opted to utilize the maximizer of tumor nanoparticle accumulation for the last treatment cycle, which maintained continuous tumor regression - similar to what could have happened by using the minimizer of tumor diameter - while gaining toxicological benefit. This protocol might be of use when treating tumors through multiple nanoparticle injections, i.e., minimizing the tumor diameter in the first few cycles until the size decreases to the point at which drug potency becomes less sensitive to nanoparticle diameter. Injection of nanoparticles with designs associated with high tumoral accumulation would then be optimal. Here, daily injection is assumed. The holiday time will be optimized in future studies.

On a patient-specific basis, the tumor model can be calibrated to fit morphological and phenotypical parameters of actual tumors after obtaining this information through imaging and histological analysis^17,57–62^. Incorporation of further biological data, such as tumor vessel density, the amount of blood perfusion, integrity of endothelial cell layer, extracellular matrix protein, interstitial pressure, infiltrated immune cells, rate of tumor cell proliferation, resistant/anti-apoptotic mechanisms in tumor cells and their heterogeneities inside the tumor, would be expected to refine the prediction of drug delivery and therapeutic potency. If drug design is to be considered, genomic characterization of mutated cells could be incorporated in an expanded tumor model. Based on the predictive model, optimization studies, similar to those presented here, would then be conducted to find the optimal nanoparticle designs with clinically-based objectives, e.g., minimization of tumor size. Practically, the optimal nanoparticle design may not be clinically available, and thus the most similar currently available configuration would need to be chosen. In this case, this particular (sub-optimal) design would need to undergo modeling evaluation to obtain a revised prediction of potency. It may also prove useful to update the predictive model according to the actual tumor response, and perform repetitive optimization studies based on the updated model to design subsequent treatment cycles. This process could be repeated before each nanoparticle treatment, taking advantage of the speed of the optimization process, until tumor remission is achieved or neoadjuvant therapy is completed.

This study complements previous efforts aimed at characterizing nanoparticles that have maximal targeting properties. Sen Gupta^29^ provided a review of computational and experimental investigations that were performed to find nanoparticle designs that enhance the margination, adhesion, and internalization process. While those studies explored designs based on evaluating finite possibilities of nanoparticle sizes and aspect ratios, finding an optimal design among a large number of candidates would only be possible through either expensive evaluation of all the possible designs or utilization of numerical optimization methods. The obtained optimal solutions in our study showed agreement with previous experimental findings. For instance, Joshi *et al*.^9^ evaluated *in vivo* the tumoral uptake of a set of liposomes differing in size, mainly {60, 80, 200, 650, 670 nm}. Among the considered sizes, they found that the maximal uptake by tumors happened with 200 nm, a size that is closest to the maximizer (147–288 nm) of tumor nanoparticle accumulation obtained in this study. In^17^, van de Ven *et al*. studied the effect of drug loading and nanoparticle concentration on tumor regression, showing that there exists a nonlinear relationship between between tumor regression and drug concentration. In this study, we have incorporated the drug potency as an optimization variable to not only couple its selection with the nanoparticle design, but also to exploit its impact for minimizing the tradeoff between tumor reduction and nanoparticle accumulation. Charoenphol *et al*.^3^ and Boso *et al*.^6^ studied the effect of nanoparticle size on the binding to vasculature walls *in vitro*, postulating that maximum binding happens with a moderate value among the sizes tested. Our results shows that the relation between nanoparticle accumulation and size is non-monotonic, i.e., agreeing with^3,6^ that the maximizer of nanoparticle binding is an interior optimum. From a therapeutic perspective, the minimizer of tumor diameter obtained in this study is 288 nm, which is very close to the 283 nm diameter nanoparticle synthesized with maximal entrapment efficiency in^8^. Not only is this size associated with a high drug load, but it is also suitable for passive tumor targeting through the EPR effect^8^. Rostami *et al*.^7^ targeted tumors derived from the SKBR3 breast cancer cell line in mice. They used multiple injections of Doxorubicin-loaded nanoparticles, showing that the rate of tumor regression decreases with tumor size. Our study is consistent with this finding, showing that the change in tumor size decreases in time following multiple treatment cycles (Fig. 7). Having to dynamically select the proper nanoparticle size as the tumor changes in response to treatment requires different optimal nanoparticle designs at the beginning of each treatment cycle, which would be possible via optimization studies such as the ones presented here.

Cancer nanotherapy faces major barriers hindering its efficacy and limiting its translation to the clinic^63^. These barriers relate not only to synthesis processes and cost, but also to biological factors such as heterogeneity of the tumor microenvironment, immune system interactions, and acquired drug resistance^64^. Heterogeneity in therapeutic efficacy has been found among various tumor types, among patients bearing same tumor types, within single patients bearing multiple metastatic tumors or even within single tumors. There also exists time- and therapy-dependent evolution in tumor response to the same therapies. This study has applied optimization as a first step towards addressing these challenges to systematically help evaluate nanoparticle therapeutic potential by maximizing drug targeting while reducing systemic toxicity. Ideally, these methods would allow for the design of nanoparticles to be customized to patient-specific tumors, while offering the capability to quickly evaluate adjustments to nanotherapy parameters during treatment. A major premise in this study is that nanoparticle-mediated drug delivery can offer substantially reduced systemic toxicity while increasing local drug delivery. Studies have shown that nanomedicine is associated with significant increases in the area under the drug concentration-time curve as compared to conventional chemotherapy^65^. This has spurred the development of nanomedications (around 9 FDA-approved and more than 30 under clinical trials^66^) to treat various types of cancer.

The analyses presented here are based on several assumptions. First, some biological parameters, such as hematocrit and drug diffusivity, were held constant. In reality, these parameters may vary by cancer type and tissue morphology and have an impact on the nanoparticle pharmacokinetics^37,67^. Future studies will use probability density functions to capture these variations and produce more robust designs that could be used for a wider range of tumors. Another source of uncertainty is variability in nanoparticle synthesis. Although optimal designs were generated in this study, it would be impractical to produce all nanoparticles equivalently, as they would be made with certain variations in their structure. Future work should account for these variations through robust optimization techniques that increase the applicability of the obtained design. In addition, tumor size at the beginning of the treatment may affect the optimal solution. Therefore, future work will study the impact of tumor size on the response. Finally, shape and functionalization have an effect on nanoparticle distribution and therapeutic potential^22,29^. This study shows that the integration of optimization in the design process makes it possible to investigate the efficiency of different nanoparticle designs, exploring new trends that may lower treatment toxicity while efficiently eradicating tumors.

## Conclusion

In this study, the design of drug-carrying nanoparticles is optimized to maximize tumor regression and minimize the treatment toxicity. Tumor regression is quantified as the percentage change of tumor diameter to that at the beginning of the treatment. The treatment toxicity is measured as the fraction of the injected nanoparticles that accumulate in the tumor. The proposed nanoparticle designs provide a basis for further experimental and computational investigations. The study sheds light on design practices that increase nanoparticle circulation time while maintaining large drug encapsulation efficiency. This work lays a foundation to quantitatively evaluate preclinical nanoparticle-based drug delivery trials and support decisions in precision medicine where optimal solutions are required on a patient-specific basis. To achieve this goal, the following are suggested to focus the translational effort of mathematical modeling and optimization of nanotherapy. Models of adequate fidelity should be selected depending on tumor characteristics and the clinical problem under consideration. For instance, local injection of nanoparticles may benefit from designs that are suitable for treating tumors locally, for which the vasculature may be used for nanoparticle injections similar to intra-arterial chemotherapy for liver cancer^68^. Possible expansion of the model to accommodate a wider type of cancers include consideration of the extracellular matrix, presence of drug resistant cells, immune system activity, molecular-scale information (e.g., genetic, and proteomic), and nanoparticle accumulation in other organs such as the spleen, kidneys, and liver. A corresponding expansion of model input parameters would be necessary to capture this information. Lastly, a clinically-relevant user interface would enable parameter input and the delivery of modeling results.

## Data Availability

All data analysed during this study are included in this published article. Additional datasets generated are available from the corresponding author upon request.

## References

[1] Martínez-Carmona Marina, Lozano Daniel, Colilla Montserrat, Vallet-Regí María (2018). Lectin-conjugated pH-responsive mesoporous silica nanoparticles for targeted bone cancer treatment. Acta Biomaterialia.

[2] Toy R, Hayden E, Shoup C, Baskaran H, Karathanasis E (2011). The effects of particle size, density and shape on margination of nanoparticles in microcirculation. Nanotechnol..

[3] Charoenphol P (2011). Targeting therapeutics to the vascular wall in atherosclerosis-carrier size matters. Atheroscler..

[4] Patil VRS, Campbell CJ, Yun YH, Slack SM, Goetz DJ (2001). Particle diameter influences adhesion under flow. Biophys. Joural.

[5] Decuzzi P., Ferrari M. (2006). The adhesive strength of non-spherical particles mediated by specific interactions. Biomaterials.

[6] Boso, D. P., Lee, S. Y., Ferrari, M., Schrefler, B. A. & Decuzzi, P. Optimizing particle size for targeting diseased microvasculature: from experiments to artificial neural networks. *Int J Nanomedicine* **6**, 1517–26 http://www.ncbi.nlm.nih.gov/pubmed/21845041. 10.2147/IJN.S20283 (2011). 10.2147/IJN.S20283 PMC3152469 21845041

[7] Rostami I (2016). Peptide-conjugated pegylated pamam as a highly affinitive nanocarrier towards her2-overexpressing cancer cells. RSC Adv..

[8] Vardhan Harsh, Mittal Pooja, Adena Sandeep Kumar Reddy, Mishra Brahmeshwar (2017). Long-circulating polyhydroxybutyrate-co-hydroxyvalerate nanoparticles for tumor targeted docetaxel delivery: Formulation, optimization and in vitro characterization. European Journal of Pharmaceutical Sciences.

[9] Joshi Shailendra, Cooke Johann R. N., Chan Darren K. W., Ellis Jason A., Hossain Shaolie S., Singh-Moon Rajinder P., Wang Mei, Bigio Irving J., Bruce Jeffrey N., Straubinger Robert M. (2016). Liposome size and charge optimization for intraarterial delivery to gliomas. Drug Delivery and Translational Research.

[10] Zhang RX (2017). Design of nanocarriers for nanoscale drug delivery to enhance cancer treatment using hybrid polymer and lipid building blocks. Nanoscale.

[11] Decuzzi P, Lee S, Bhushan B, Ferrari M (2005). A theoretical model for the margination of particles within blood vessels. Annals Biomed. Eng..

[12] Cristini Vittorio, Lowengrub John, Nie Qing (2003). Nonlinear simulation of tumor growth. Journal of Mathematical Biology.

[13] McDougall SR, Anderson ARA, Chaplain MAJ (2006). Mathematical modelling of dynamic adaptive tumour-induced angiogenesis: Clinical implications and therapeutic targeting strategies. J. Theor. Biol..

[14] Macklin Paul, McDougall Steven, Anderson Alexander R. A., Chaplain Mark A. J., Cristini Vittorio, Lowengrub John (2008). Multiscale modelling and nonlinear simulation of vascular tumour growth. Journal of Mathematical Biology.

[15] Wu Min, Frieboes Hermann B., McDougall Steven R., Chaplain Mark A.J., Cristini Vittorio, Lowengrub John (2013). The effect of interstitial pressure on tumor growth: Coupling with the blood and lymphatic vascular systems. Journal of Theoretical Biology.

[16] Frieboes HB, Wu M, Lowengrub J, Decuzzi P, Cristini V (2013). A computational model for predicting nanoparticle accumulation in tumor vasculature. PLoS ONE.

[17] van de Ven Anne L., Wu Min, Lowengrub John, McDougall Steven R., Chaplain Mark A. J., Cristini Vittorio, Ferrari Mauro, Frieboes Hermann B. (2012). Integrated intravital microscopy and mathematical modeling to optimize nanotherapeutics delivery to tumors. AIP Advances.

[18] Wu, M. *et al*. The effect of interstitial pressure on therapeutic agent transport: coupling with the tumor blood and lymphatic vascular systems. *J Theor Biol* **355**, 194–207, http://www.ncbi.nlm.nih.gov/pubmed/2475192710.1016/jjtbi.2014.04.012 (2014). 10.1016/j.jtbi.2014.04.012 PMC4098870 24751927

[19] Curtis Louis T., Wu Min, Lowengrub John, Decuzzi Paolo, Frieboes Hermann B. (2015). Computational Modeling of Tumor Response to Drug Release from Vasculature-Bound Nanoparticles. PLOS ONE.

[20] England CG, Ng CF, van Berkel V, Frieboes HB (2015). A review of pharmacological treatment options for lung cancer: Emphasis on novel nanotherapeutics and associated toxicity. Curr. Drug Targets.

[21] Curtis Louis T., Frieboes Hermann B. (2016). The Tumor Microenvironment as a Barrier to Cancer Nanotherapy. Advances in Experimental Medicine and Biology.

[22] Chamseddine Ibrahim M., Kokkolaras Michael (2018). Nanoparticle Optimization for Enhanced Targeted Anticancer Drug Delivery. Journal of Biomechanical Engineering.

[23] Decuzzi Paolo, Pasqualini Renata, Arap Wadih, Ferrari Mauro (2008). Intravascular Delivery of Particulate Systems: Does Geometry Really Matter?. Pharmaceutical Research.

[24] Truong Nghia P, Whittaker Michael R, Mak Catherine W, Davis Thomas P (2014). The importance of nanoparticle shape in cancer drug delivery. Expert Opinion on Drug Delivery.

[25] Sikkandhar Musafar, Nedumaran Anu, Ravichandar Roopa, Singh Satnam, Santhakumar Induja, Goh Zheng, Mishra Sachin, Archunan Govindaraju, Gulyás Balázs, Padmanabhan Parasuraman (2017). Theranostic Probes for Targeting Tumor Microenvironment: An Overview. International Journal of Molecular Sciences.

[26] Le Digabel S (2011). Algorithm 909: Nomad: Nonlinear optimization with the mads algorithm. ACM Transactions on Math. Softw..

[27] Audet C, Dennis J (2006). Mesh adaptive direct search algorithms for constrained optimization. SIAM J. on Optim..

[28] Audet C, Kokkolaras M, Le Digabel S, Talgorn B (2018). Order-based error for managing ensembles of surrogates in mesh adaptive direct search. J. Glob. Optim..

[29] Sen Gupta Anirban (2015). Role of particle size, shape, and stiffness in design of intravascular drug delivery systems: insights from computations, experiments, and nature. Wiley Interdisciplinary Reviews: Nanomedicine and Nanobiotechnology.

[30] Hermann P., Armant M., Brown E., Rubio M., Ishihara H., Ulrich D., Caspary R.G., Lindberg F.P., Armitage R., Maliszewski C., Delespesse G., Sarfati M. (1999). The Vitronectin Receptor and its Associated CD47 Molecule Mediates Proinflammatory Cytokine Synthesis in Human Monocytes by Interaction with Soluble CD23. The Journal of Cell Biology.

[31] Wang L, Pan D, Yan Q, Song Y (2017). Activation mechanisms of anb3 integrin by binding to fibronectin: A computational study. Protein Sci..

[32] Yokoyama K, Zhang X-P, Medved L, Takada Y (1999). Specific binding of integrin anb3 to the fibrinogen y and ae chain c-terminal domains. Biochem..

[33] Kang Y-J, Forbes K, Carver J, Aplin JD (2014). The role of the osteopontin-integrin anb3 interaction at implantation: functional analysis using three different *in vitro* models. Hum. Reproduction.

[34] Bertsimas D, Brown DB, Caramanis C (2011). Theory and applications of robust optimization. SIAM Rev..

[35] Yang W (2013). Genomics of drug sensitivity in cancer (gdsc): a resource for therapeutic biomarker discovery in cancer cells. Nucleic Acids Res..

[36] Lophaven, S., Nielsen, H. B. & Sondergaard, J. Dace – a matlab kriging toolbox (2002).

[37] Sims, L. B., Huss, M. K., Frieboes, H. B. & Steinbach-Rankins, J. M. Distribution of PLGA-modified nanoparticles in 3D cell culture models of hypo-vascularized tumor tissue. *J Nanobiotechnology* **15**, 67, http://www.ncbi.nlm.nih.gov/pubmed/28982361. 10.1186/s12951-017-0298-x (2017). 10.1186/s12951-017-0298-x PMC5629750 28982361

[38] Curtis Louis T., Rychahou Piotr, Bae Younsoo, Frieboes Hermann B. (2016). A Computational/Experimental Assessment of Antitumor Activity of Polymer Nanoassemblies for pH-Controlled Drug Delivery to Primary and Metastatic Tumors. Pharmaceutical Research.

[39] Hickey John W., Santos Jose Luis, Williford John-Michael, Mao Hai-Quan (2015). Control of polymeric nanoparticle size to improve therapeutic delivery. Journal of Controlled Release.

[40] Kadian RN (2018). A promising drug delivery approach. Asian J. Pharm. Clin. Res..

[41] Cao Shijie, Jiang Yonghou, Levy Claire N., Hughes Sean M., Zhang Hangyu, Hladik Florian, Woodrow Kim A. (2018). Optimization and comparison of CD4-targeting lipid-polymer hybrid nanoparticles using different binding ligands. Journal of Biomedical Materials Research Part A.

[42] Lima Ana Catarina, Alvarez-Lorenzo Carmen, Mano João F. (2016). Design Advances in Particulate Systems for Biomedical Applications. Advanced Healthcare Materials.

[43] Guo Dandan, Shi Changying, Wang Xu, Wang Lili, Zhang Shengle, Luo Juntao (2017). Riboflavin-containing telodendrimer nanocarriers for efficient doxorubicin delivery: High loading capacity, increased stability, and improved anticancer efficacy. Biomaterials.

[44] Schmid Günter, Kreyling Wolfgang G., Simon Ulrich (2017). Toxic effects and biodistribution of ultrasmall gold nanoparticles. Archives of Toxicology.

[45] Foged C, Brodin B, Frokjaer S, Sundblad A (2005). Particle size and surface charge affect particle uptake by human dendritic cells in an *in vitro* model. Int. J. Pharm..

[46] Gratton SEA (2008). The effect of particle design on cellular internalization pathways. Proc. Natl. Acad. Sci..

[47] Muro S (2008). Control of endothelial targeting and intracellular delivery of therapeutic enzymes by modulating the size and shape of icam-1-targeted carriers. Mol. Ther..

[48] Li Lei, Zhao Xiuli, Yang Chunrong, Hu Haiyang, Qiao Mingxi, Chen Dawei (2011). Preparation and optimization of doxorubicin-loaded albumin nanoparticles using response surface methodology. Drug Development and Industrial Pharmacy.

[49] Elsaid Ali AA, Taher M, Mohamed F (2013). Microencapsulation of alpha-mangostin into PLGA microspheres and optimization using response surface methodology intended for pulmonary delivery. J. Microencapsulation.

[50] Asghar S (2014). A facile approach for crosslinker free nano self assembly of protein for anti-tumor drug delivery: Factors’ optimization, characterization and *in vitro* evaluation. Eur. J. Pharm. Sci..

[51] Chaubey Pramila, Patel Ravi R, Mishra Brahmeshwar (2014). Development and optimization of curcumin-loaded mannosylated chitosan nanoparticles using response surface methodology in the treatment of visceral leishmaniasis. Expert Opinion on Drug Delivery.

[52] Boonyasirisri P, Nimmannit U, Rojsitthisak P, Bhunchu S, Rojsitthisak P (2015). Optimization of curcuminoidloaded PLGA nanoparticles using box-behnken statistical design. J. Nano Res..

[53] Akl MA (2016). Factorial design formulation optimization and *in vitro* characterization of curcumin-loaded PLGA nanoparticles for colon delivery. J. Drug Deliv. Sci. Technol..

[54] de Oliveira Pedro Rafael, Goycoolea Francisco M., Pereira Susana, Schmitt Carla C., Neumann Miguel G. (2018). Synergistic effect of quercetin and pH-responsive DEAE-chitosan carriers as drug delivery system for breast cancer treatment. International Journal of Biological Macromolecules.

[55] Rajpoot Kuldeep, Jain Sunil K. (2017). Colorectal cancer-targeted delivery of oxaliplatin via folic acid-grafted solid lipid nanoparticles: preparation, optimization, and in vitro evaluation. Artificial Cells, Nanomedicine, and Biotechnology.

[56] Brannon-Peppas Lisa, Blanchette James O. (2004). Nanoparticle and targeted systems for cancer therapy. Advanced Drug Delivery Reviews.

[57] Frieboes HB (2006). An integrated computational/experimental model of tumor invasion. Cancer Res..

[58] Frieboes HB (2007). Computer simulation of glioma growth and morphology. Neuroimage.

[59] Frieboes HB (2009). Prediction of drug response in breast cancer using integrative experimental/computational modeling. Cancer Res..

[60] Frieboes HB (2013). An integrated computational/experimental model of lymphoma growth. PLOS Comput. Biol..

[61] van de Ven Anne L, Abdollahi Behnaz, Martinez Carlos J, Burey Lacey A, Landis Melissa D, Chang Jenny C, Ferrari Mauro, Frieboes Hermann B (2013). Modeling of nanotherapeutics delivery based on tumor perfusion. New Journal of Physics.

[62] Frieboes HB (2015). Predictive modeling of drug response in non-hodgkin’s lymphoma. PLOS ONE.

[63] Desai N (2012). Challenges in development of nanoparticle-based therapeutics. The AAPS J..

[64] Wilhelm S (2016). Analysis of nanoparticle delivery to tumours. Nat. Rev. Mater..

[65] Gabizon A, Shmeeda H, Barenholz Y (2003). Pharmacokinetics of pegylated liposomal doxorubicin. Clin. Pharmacokinet..

[66] Hare JI (2017). Challenges and strategies in anti-cancer nanomedicine development: An industry perspective. Adv. Drug Deliv. Rev..

[67] Sims, L. B. *et al*. Efficacy of surface-modified PLGA nanoparticles to treat cervical cancer (in press) (2018). 10.1007/s11095-019-2602-y PMC7271733 30868271

[68] Spada, F. *et al*. Hepatic intra-arterial chemotherapy in patients with advanced primary liver tumours. *Ecancermedicalscience* **6**, 10.3332/ecancer.2012.280 (2012). 10.3332/ecancer.2012.280 PMC3512295 23226162

